# Heterogeneity in the Paraventricular Thalamus: The Traffic Light of Motivated Behaviors

**DOI:** 10.3389/fnbeh.2020.590528

**Published:** 2020-10-16

**Authors:** Jacqueline F. McGinty, James M. Otis

**Affiliations:** Department of Neuroscience, Medical University of South Carolina, Charleston, SC, United States

**Keywords:** paraventricular thalamic nucleus, chemogenetic, heterogeneity, hypothalamus, optogenetic, prefrontal cortex, reward networks

## Abstract

The paraventricular thalamic nucleus (PVT) is highly interconnected with brain areas that control reward-seeking behavior. Despite this known connectivity, broad manipulations of PVT often lead to mixed, and even opposing, behavioral effects, clouding our understanding of how PVT precisely contributes to reward processing. Although the function of PVT in influencing reward-seeking is poorly understood, recent studies show that forebrain and hypothalamic inputs to, and nucleus accumbens (NAc) and amygdalar outputs from, PVT are strongly implicated in PVT responses to conditioned and appetitive or aversive stimuli that determine whether an animal will approach or avoid specific rewards. These studies, which have used an array of chemogenetic, optogenetic, and calcium imaging technologies, have shown that activity in PVT input and output circuits is highly heterogeneous, with mixed activity patterns that contribute to behavior in highly distinct manners. Thus, it is important to perform experiments in precisely defined cell types to elucidate how the PVT network contributes to reward-seeking behaviors. In this review, we describe the complex heterogeneity within PVT circuitry that appears to influence the decision to seek or avoid a reward and point out gaps in our understanding that should be investigated in future studies.

## Introduction

Several recent reviews have comprehensively summarized the literature on the role of the paraventricular nucleus of the thalamus (PVT) in reward-seeking (James and Dayas, 2013; Kirouac, 2015; Do-Monte and Kirouac, 2017; Millan et al., 2017; Zhou and Zhu, 2019; Matzeu and Martin-Fardon, 2020). Based on a growing body of evidence, a consensus is emerging that PVT is part of a network that mediates appetitive/approach and aversive/avoidance behaviors, particularly during motivational conflicts (Choi and McNally, 2017; Choi et al., 2019). Other studies suggest that PVT exerts a categorically-free modulatory influence on downstream limbic targets signaling salience but not valence (Beas et al., 2018; Zhu et al., 2018). How PVT contributes to both positively- and negatively-valenced behaviors appears to rely on neuronal subpopulations that are characterized by several criteria: (1) different locations within PVT; (2) distinct connections; (3) differentially-expressed genes; and (4) distinct activity-related profiles. However, up until now, efforts to investigate the role of PVT subpopulations based on these criteria in appetitive/approach and aversive/avoidance behaviors have been limited. In this review, we point out areas in which conflicting or inconsistent conclusions about PVT functioning may be resolved by combining existing and emerging technology to define the characteristics and function of PVT cell populations.

## Global PVT Manipulation Leads to Mixed Behavioral Outcomes

Classic and conventional experimental approaches aimed to globally modulate PVT activity during reward-seeking and consumption have resulted in mixed, and even opposing, behavioral outcomes, clouding our understanding of how exactly PVT influences appetitive behaviors. Initial studies demonstrated that lesions or pharmacological inhibition of PVT lead to increased food-seeking and consumption (Bhatnagar and Dallman, 1999; Stratford and Wirtshafter, 2013; Haight et al., 2015), although others have found that the same manipulations have no effect on those behaviors or that the behavioral effects rely heavily on physiological and experimental conditions (Nakahara et al., 2004; Landry et al., 2007; Haight et al., 2015; Do-Monte et al., 2017). Furthermore, while one study suggests that PVT is essential for behavioral and physiological anticipation of food based on an animal’s circadian rhythms (Nakahara et al., 2004), others find no effect of PVT ablation on these anticipatory phenomena (Landry et al., 2007). Overall, broad manipulations of PVT neuronal activity have led to diverse effects on food reward-related behaviors.

Studies investigating the influence of PVT on drug-related phenomena have also described profoundly diverse behavioral outcomes. Lesions or pharmacological inhibition of PVT enhance locomotor responses to cocaine (Young and Deutch, 1998) but also suppress cocaine conditioned locomotor sensitization (Young and Deutch, 1998), cocaine conditioned place preference expression (Browning et al., 2014), and cue- and drug-induced reinstatement of cocaine-seeking (James et al., 2010; Matzeu et al., 2015; Wunsch et al., 2017). In contrast, transient inactivation of PVT enhances cue-induced reinstatement after extinction in goal-tracking, but not sign-tracking, rats (Kuhn et al., 2018). The mid-portion (James et al., 2010; Matzeu et al., 2015) or the entire anterior-posterior extent of PVT (Kuhn et al., 2018) was targeted in these studies. Furthermore, global lesions of PVT do not affect alcohol self-administration but reduce context-induced alcohol-seeking (Hamlin et al., 2009). Finally, pharmacological or chemogenetic inactivation of PVT does not affect heroin-seeking behavior whereas chemogenetic activation of PVT suppresses heroin seeking induced by chronic food restriction (Chisolm et al., 2020). As we learn more about PVT heterogeneity, it is important to consider that a lack of specificity in targeting PVT subpopulations may have contributed to disparate findings and conclusions in the literature. Overall, studies using classic and more recent, now conventional, experimental approaches highlight the complexity of PVT and suggest that greater experimental specificity may increase our understanding of how PVT controls reward-related behaviors.

## PVT Heterogeneity Gates The Motivational Properties of Approach-Avoidance Decisions

Different degrees of food palatability and content as well as the availability of food under different conditions of hunger and risk determine PVT’s contributions to food-seeking and consumption. These findings suggest that PVT gates the motivation to seek food, for example, depending on how hungry the animal is and whether it faces conditions that signal relative safety (GO) vs. risk (STOP) in foraging for food. Substance use disorders also can be characterized as an approach (GO)-avoidance (STOP) problem. The transition to addiction evolves from the initial euphoric responses to addictive drugs to repeated drug use and maladaptive behaviors aimed to relieve the dysphoria associated with drug abstinence. The compulsion to seek and take drugs eventually overwhelms the motivation to abstain despite negative consequences. Recent evidence has implicated posterior PVT in this motivation (Giannotti et al., 2018).

Whether PVT is activated or inhibited as a consequence of reward-seeking depends on the location, gene transcriptional profile, activity, and projections of the neuronal subpopulations sampled, as well as on the method of measuring “activity.” Induction of the immediate early gene, *c-fos*, or its encoded protein, Fos, is commonly considered a marker of neuronal activity that has been used in reward-seeking studies and is most notably expressed following the presentation of food-predictive cues (Flagel et al., 2011). Addictive drugs, whether experimenter- or self-administered, and conditioned cues that are associated with addictive drugs also have been reported to increase global PVT Fos expression (summarized in Table 3 in Millan et al., 2017). However, this Fos response is not universal among PVT neurons and the identity of the Fos-positive neurons has largely been uncharacterized, masking their potential heterogeneity under different conditions of drug-taking and seeking. Moreover, Fos expression is an indirect measurement that has several limitations. First, Fos expression does not reflect activity time-locked to behavior because Fos transcription and translation take time; second, basal expression of Fos is very low and thus Fos expression is a poor indicator of reduced neuronal activity. More recent studies using approaches that detect single-cell dynamics, such as unit recordings and calcium imaging, have reported both excitatory (Labouèbe et al., 2016; Cheng et al., 2018) and inhibitory (Do-Monte et al., 2017; Zhang and van den Pol, 2017; Otis et al., 2019) signals in PVT neurons in response to food-seeking under various physiological and environmental conditions. These approaches highlight the heterogenous response dynamics in PVT cell populations and suggest that distinct classes of PVT neurons may differentially regulate motivated behaviors (see “Heterogeneous Activity Dynamics in PVT Neurons” section below).

## Projection-Specific Pathways Originating in PVT Regulate Different Motivated Behaviors

### Different Areas of PVT Project to Different Targets That Regulate Emotionally-Valent Behaviors

Anterior (a)PVT has been linked to appetitive/approach behaviors and posterior (p)PVT to aversive/avoidance behaviors (Bhatnagar et al., 2003; Li et al., 2014; Matzeu et al., 2015; Do-Monte et al., 2017). Valence-dependency may be explained by aPVT and pPVT projections to different subdivisions of reward circuitry: aPVT projects predominantly to the dorsomedial nucleus accumbens (NAc) shell and ventral subiculum whereas pPVT projects predominantly to the ventromedial NAc shell, central amygdala (CeA), and bed nucleus of the stria terminalis (BNST; Li and Kirouac, 2008; Vertes and Hoover, 2008). Photostimulation of dynorphin-positive neurons in the dorsomedial NAc shell causes a real-time place preference whereas photostimulation of dynorphin-positive neurons in the ventromedial shell produces a real-time place aversion (Al-Hasani et al., 2015). Also, a coordinated reduction of excitatory input to the anterior, and an increase in input to the posterior, NAc shell, from the basolateral amygdala (BLA), ventral hippocampus/subiculum, and the midline thalamus is sufficient to induce reward-related feeding (Reed et al., 2018). These data reveal that activity in excitatory afferents to the NAc shell is characterized more accurately by their anterior-posterior termination in the NAc than by the origin of the afferent fibers. Together these studies emphasize the need to carefully target and map downstream subregions from PVT to obtain a complete functional picture of the circuitry regulating different motivated behaviors.

### Physiological and Environmental Conditions Determine the Role of PVT→NAc Activity in Reward-Seeking and Consumption

The type and strength of environmental stimuli also have a major impact on PVT gating of ingestive behaviors. For example, Labouèbe et al. (2016) reported that photostimulation of glucose transporter 2 (GluT2)-positive PVT→NAc shell neurons or knockdown of GluT2 increases sucrose seeking. These GluT2^+^ PVT→NAc shell neurons are profoundly sensitive to low glucose levels that motivate sucrose seeking and consumption (Labouèbe et al., 2016). This subpopulation of PVT→NAc neurons that increases sucrose seeking when photostimulated contrasts with aPVT→NAc neurons that increase sucrose seeking when photoinhibited (Do-Monte et al., 2017). However, the inhibition of the latter population only increased seeking when a sucrose reward unexpectedly was omitted but not when it was available (Do-Monte et al., 2017; Lafferty et al., 2020), suggesting that aPVT→NAc shell neurons mediate behavioral suppression when reward-seeking would be unproductive. These studies indicate that even within the PVT→NAc shell pathway, there is heterogeneity and differential regulation of reward-related behaviors depending on the particular conditions and neuronal subpopulations under investigation. Consistent with this interpretation, photostimulation of the aPVT→NAc shell pathway promoted food-seeking when rats were presented with food in a novel (potentially unsafe) environment (Cheng et al., 2018). In this study, when the aPVT→NAc shell pathway was stimulated, fasted rats increased the time spent feeding in a lighted center area of an open field but not when fed in a familiar home cage. Moreover, feeding in the novel environment was delayed related to the onset of photostimulation, suggesting that PVT dynamically regulates whether to approach or avoid food depending on environmental conditions. Altogether, these findings suggest that a greater definition of state-dependence and environmental conditions is needed to fully understand the modulatory role of PVT projection neurons in reward-related behaviors.

### Physiological Conditions Determine the Role of PVT→NAc Neurons for Drug-Associated Memory and Withdrawal

Of the few studies that have evaluated PVT responses during withdrawal in dependent animals, Smith et al. (2019) reported a persistent decrease in Fos-positive neurons in PVT (among other brain regions) that persisted after 7 days of withdrawal from chronic intermittent alcohol vapor exposure in dependent mice, but these neurons were not further characterized. In contrast, Zhu et al. (2016) reported Fos induction in PVT→NAc neurons during naloxone-precipitated morphine withdrawal in mice. They went on to show that photostimulation of the PVT→NAc pathway caused a real-time place aversion and photoinhibition of this pathway blocked the aversive and the somatic effects of naloxone-precipitated opioid withdrawal. More recently, this group showed that PVT→NAc shell photoinhibition blocked retrieval (but not acquisition) of morphine-associated memories and morphine-primed relapse in a morphine-conditioned place preference assay (Keyes et al., 2020). These findings that activation of PVT→NAc neurons underlies both withdrawal and drug-associated memory expression are surprising and suggest that distinct physiological conditions can determine how activity in this pathway influences behavior. Interestingly, Keyes et al. (2020) also found that the effects of PVT→NAc inhibition on opioid-associated memory retrieval were long-lasting whereas inhibition of the neurons immediately after retrieval (during memory reconsolidation) had no effect. These behavioral effects are similar to optogenetic studies revealing long-lasting impairment of fear memories (Do-Monte et al., 2015) and sucrose-associated memories (Otis et al., 2017) when photo modulating PVT circuit activity during, but not immediately after, cue exposure. These findings could be related specifically to adaptations in cortico-thalamo-striatal circuitry, as extensive datasets reveal that inhibition of neuronal excitability in the prelimbic area of the medial prefrontal cortex (PFC) can persistently impair retrieval and relapse in a cocaine-associated memory paradigm without affecting memory reconsolidation (Otis et al., 2013, 2018; Otis and Mueller, 2017). Furthermore, these effects are likely related to top-down modulation of PVT→NAc neurons, as photomodulation of prefrontal inputs to PVT→NAc neurons persistently impairs sucrose-associated memories and downstream activity in PVT→NAc neurons (Otis et al., 2017). Regardless, more studies are required to fully understand how cortico-thalamo-striatal circuitry may contribute to the maintenance of these reward-related memories.

### Environmental Conditions Determine the Role of PVT→CeA Activity for Reward-Seeking

In contrast to inducing sucrose seeking in the absence of reward by photoinhibiting the PVT→NAc shell pathway, aPVT→CeA photoinhibition decreased sucrose seeking when the sucrose reward was omitted (Do-Monte et al., 2017). The authors concluded that activating PVT→CeA neurons increases negative affect (reward omission-induced frustration). Consistent with this interpretation, photoinhibition of PVT→CeA neurons during memory consolidation decreased the expression of fear-conditioned freezing behavior (Do-Monte et al., 2015; Penzo et al., 2015). Further, a somewhat surprising role for the PVT→CeA pathway comes from a more recent report indicating that photoinhibition of PVT→CeA neurons decreases the formation of a morphine conditioned place preference, suggesting that this pathway associates opiate reward to the context in which it is experienced (Keyes et al., 2020). The different conditions that engage PVT→CeA and/or PVT→NAc shell neurons may be coordinated by PVT neurons that project to both regions individually or by the subpopulation of PVT neurons that collateralize to both CeA and NAc; approximately 7.7% of aPVT neurons and 8.4% of pPVT neurons that project to CeA also project to NAc shell, as described by Dong et al. (2017).

## Distinct Inputs to PVT Regulate Different Motivated Behaviors

Discrete PVT pathways enable approach or avoidance behaviors that are selected based on the type and strength of afferent input they receive. What do we know about the functional distinctions between these inputs? Generally, PVT receives convergent inputs from widespread areas of the brainstem that primarily mediate visceral functions, from the hypothalamus that mediate arousal and homeostatic functions, and from the neocortex that contribute to learning and attention (reviewed in Kirouac, 2015). Ascending monoaminergic inputs from the brainstem to PVT are dense; surprisingly, projections from the locus coeruleus release dopamine as well as norepinephrine in PVT (Beas et al., 2018). Peptidergic neurons that also release glutamate and/or GABA arise primarily from hypothalamic areas and also provide particularly strong innervation to all regions of PVT (as reviewed in this thematic series). Finally, excitatory glutamatergic neurons from the medial PFC also innervate PVT, although their functional control of PVT activity is not quite as profound or widespread as that of hypothalamic inputs (Otis et al., 2019). Below, we discuss the influence of these highly-convergent inputs to PVT on reward-seeking.

### Hypothalamic and Peri-hypothalamic Inputs and Reward-Seeking

The PVT forms a link between the hypothalamus and the striatum that is critical for reward-seeking and that is particularly well-documented with regard to hedonic feeding behaviors (Kelley et al., 2005). Orexin/hypocretin-positive neurons from the perifornical-lateral hypothalamic area (LHA) form a dense axonal plexus in PVT (Peyron et al., 1998; Kelley et al., 2005). These projections are thought to contribute arousal and metabolic information to PVT that influences palatable food-seeking in both hungry and sated rats. Systemic administration of an orexin-1 receptor antagonist decreases cue-induced food-seeking (Cole et al., 2015), and infusion of an orexin-1 receptor shRNA into the middle anterior-posterior extent of PVT decreases palatable food consumption (hedonic feeding) in sated rats (Choi et al., 2012). Cue-induced anticipation of palatable food induces Fos in PVT neurons that are orexin receptor 1-positive, suggesting that palatable food cues activate these neurons (Choi et al., 2010). However, an orexin-1 receptor antagonist also increases Fos expression in the anterior PVT of sated rats (Cole et al., 2015), suggesting that orexin inhibits PVT neurons. It would be important to identify what subpopulations of PVT neurons respond in opposite directions to palatable food cues to understand this discrepancy. Some insight is provided by the finding that orexin’s excitatory effect on PVT activity may be indirectly mediated by orexin-induced excitation of a thalamocortical loop (Huang et al., 2006). The response of PVT neurons to orexin may also be state-dependent. Indeed, Meffre et al. (2019) reported that satiety decreases the ability of palatable food cues to excite pPVT and NAc core neurons and that the dampening of the cue-induced reward signal in sated rats is reversed by intra-PVT orexin infusion or photostimulation of pPVT→NAc core neurons. PVT neurons express both orexin-1 and orexin-2 receptors (Marcus et al., 2001) that have been differentially implicated in reward-seeking. Orexin-2, but not orexin-1, receptor levels in PVT are increased by ethanol drinking and local infusions of an Orexin-2, but not an Orexin-1, receptor antagonist reduces alcohol drinking. Orexin-2 receptors in the PVT also appear to mediate cocaine seeking (Matzeu et al., 2016), consistent with the finding that orexin-1 receptors in PVT do not (James et al., 2011b). Thus, identifying the unique characteristics of PVT neuronal populations that express orexin-1 vs. orexin-2 receptors, such as their ground-truth activity dynamics, functions, and projection profiles, merits further attention.

In contrast to orexin projections to PVT that co-release glutamate (Ishibashi et al., 2005; Huang et al., 2006), perifornical LHA neurons, AGRP^+^ neurons in the mediobasal (arcuate) hypothalamic area, and zona incerta (ZI) neurons dorsal to LHA send GABAergic projections to PVT (Li and Kirouac, 2012; Betley et al., 2013; Zhang and van den Pol, 2017). Photostimulation of ZI→PVT evokes more feeding that is shorter in onset than ARC^AGRP^→PVT activation and longer in duration than stimulation of LHA^GABA^→PVT; indeed, repeated ZI^GABA^→PVT activation evokes hyperphagia and weight gain (Zhang and van den Pol, 2017). In contrast, these authors showed that photostimulation of vGlut2-positive (excitatory) parasubthalamic nucleus projections to PVT decreased feeding. Since LHA also sends glutamatergic input to PVT→NAc neurons (Otis et al., 2019), how activation of GABA, glutamate, and neuropeptide-specific inputs to PVT differentially regulate reward-seeking and consumption requires further study using multiple cell-type-specific recording and manipulation strategies. Altogether, these studies emphasize the need to study polysynaptic PVT circuits to draw accurate conclusions about PVT’s role in food-seeking.

### Prefrontal Inputs and Reward-Seeking

The prelimbic medial PFC→PVT pathway modulates motivationally valent behaviors by establishing and maintaining responses to conditioned cues (Otis et al., 2017, 2019). In the Otis studies, photostimulation of PFC inputs to PVT→NAc neurons during the presentation of a conditioned stimulus (CS^+^) suppressed the expression of conditioned sucrose seeking and cue discrimination. The PFC→PVT role in conditioned cue-induced behavior is reinforced by a report that photoinhibition of PFC→PVT (or PVT→CeA) neurons decreased retrieval of a fear-conditioned memory from 24 h to 7 days after experiencing a foot shock paired with a tone CS^+^ (Do-Monte et al., 2015; Chen and Bi, 2019). Concerning abused drugs, transient chemogenetic inactivation of PFC→pPVT neurons immediately after the end of cocaine self-administration inhibited context-induced cocaine-seeking after 7 days of abstinence and cue-induced reinstatement of cocaine-seeking after extinction (Giannotti et al., 2018). The latter study is consistent with a recent report in which transient inhibition of PFC→PVT neurons at the time of reinstatement testing inhibited cue-induced cocaine-seeking in sign-tracking, but not goal-tracking, rats (Kuhn et al., 2020). Thus, the weight of evidence suggests that conditioned cues associated with appetitive (food, cocaine) or aversive (footshock) stimuli activate different sets of PVT neurons through inhibition or excitation of particular PFC input neurons and that the combined activity is necessary for conditioned cues to elicit reward-seeking or avoidance.

## Heterogeneous Cell Types in PVT

The PVT consists of vastly heterogeneous neurons, dissociable based on a variety of non-exclusive characteristics including anatomical location, gene expression, circuit connectivity, and activity dynamics. The combination of heterogeneous features has made it difficult to monitor and manipulate the activity of precisely defined PVT cell populations, which can have non-overlapping and even opposing functions for behavior (see above). Thus, here we discuss the cellular diversity found in PVT and highlight approaches that could be particularly useful for targeting and dissociating distinct PVT cell types.

### Heterogeneity Is Based on Anatomical Location, Projection Profile, and Gene Expression

The PVT consists of strikingly unique cell populations based on its anterior-posterior, medial-lateral, and dorsal-ventral axes. Although PVT consists of glutamatergic excitatory projection neurons that bifurcate to several downstream targets, aPVT projection neurons are thought to provide stronger innervation of PFC and ventral subiculum, whereas pPVT projection neurons provide stronger input to the BNST and CeA (Li and Kirouac, 2008). Furthermore, neurons projecting to the CeA are generally found dorsal to non-CeA projecting neurons, such as those that project to the BLA and some extent those that project to the NAc core and shell (Penzo et al., 2015; Dong et al., 2017). Finally, collateralizing neurons that project throughout the extended amygdala, including BNST, NAc shell, and/or CeA, are laterally located in PVT (Dong et al., 2017; Otis et al., 2019), whereas PFC projecting neurons may have less bias for medial or lateral PVT (Hoover and Vertes, 2007). Despite some unique efferent patterns among distinct PVT projections neurons, little is known regarding the exact gene expression characteristics of each of those cell populations.

PVT consists of separable populations of neurons based on gene expression characteristics, with some of those populations enriched in particular regions of PVT. For example, neurons in aPVT are more likely to express dopamine D1 receptors, galanin, neurotensin, and neurotrophin receptor kinase 1 as compared with neurons in pPVT. In contrast, neurons in pPVT are more likely to express dopamine D2 receptors (Gao et al., 2020; Allen Brain Gene Search). Importantly, the transition from aPVT to pPVT is not obvious or sudden, as unique gene expression characteristics and projection dynamics among PVT neurons transition gradually along the anterior-posterior gradient rather than suddenly based on particular coordinates. This has led to the proposal that the classic nomenclature of “anterior” and “posterior” subregions of PVT should be revised altogether (Gao et al., 2020). In addition to this anterior-posterior gradient, PVT neurons also express unique genes based on the medial-lateral axis, although such diversity has been less extensively studied due to difficulty in selectively targeting medial vs. lateral PVT. Recent elegant work by Gao et al. (2020) used multiplexed RNAscope to show that galanin-expressing neurons are located in medial PVT as compared with more lateral D2-expressing neurons, with very little overlap between these two cell types. Further studies such as these are critical for further dissociating distinct cell populations in PVT.

Expression of several genes, besides those coding for D2 receptors and galanin, can serve as markers for PVT but have not been dissociated from other nearby cell types. For example, two genes that are highly important for feeding, calretinin, and glucose transporter 2, are highly expressed in PVT. Calretinin, also known as calbindin 2, serves as one of the best markers for PVT (Allen Brain Atlas; Matyas et al., 2018), with around 62% of PVT glutamate neurons expressing this gene (Hua et al., 2018) predominantly in the lateral ventral and posterior regions (Winsky et al., 1992). These neurons have highly non-selective efferent projections to NAc, BNST, CeA, and PFC (Hua et al., 2018), and yet how calretinin-expressing neurons in PVT may differ from non-calretinin neurons is largely unexplored. A large population of PVT neurons also expresses a gene encoding the glucose transporter GluT2 (*Scl2a2*), and these neurons also project to the NAc (Labouèbe et al., 2016). However, little else is known about these GluT2^+^ cells, including their potential co-expression of calretinin. PVT is also a locus of peptides and peptide receptors, such as orexin receptors 1 and 2 (Marcus et al., 2001), and dense opioid receptor labeling, particularly kappa and mu-opioid receptors (Mansour et al., 1994). Electrophysiological studies confirm that PVT neurons respond to kappa and mu-opioid receptor stimulation (Chen et al., 2015) and yet the characteristics and function of opioid receptor-expressing cells in PVT are largely unknown.

Overall, these studies highlight the heterogeneity of PVT neurons based on anatomical location, projection profile, and most notably gene expression—although we have barely pierced the surface into understanding the unique cell types that exist in PVT. Due to the complexity of projection neurons in this brain region, we propose that researchers begin to dissect these cell types based on gene expression, as recently documented (Gao et al., 2020). Most importantly, high-throughput single-cell sequencing studies should be performed on PVT to identify highly unique gene expression patterns among distinct PVT cell types (Mocosko et al., 2015; Rossi et al., 2019). These studies could then be followed up with a cre-driver mouse or rat lines for targeting unique cell types that may have non-overlapping activity dynamics and functions for behavior (Witten et al., 2011).

### Heterogeneous Activity Dynamics in PVT Neurons

Single-cell recordings in PVT reveal the heterogeneous nature of PVT activity dynamics *in vivo* and highlight our need to record from genetically-distinct, precisely-defined cell populations with single-cell resolution. Several initial studies demonstrated that presentation of various reward-related stimuli—particularly alcohol, drugs, or related cues—induce the expression of immediate early genes in PVT neurons (Brown et al., 1992; Deutch et al., 1998; Rhodes et al., 2005; Dayas et al., 2008; James et al., 2011a; Matzeu et al., 2015; Zhu et al., 2016; Pelloux et al., 2018), although the specificity of this expression for distinct PVT cell types is unclear. Furthermore, how such gene expression relates to ground-truth activity dynamics among PVT neurons is unknown, a point that has recently been highlighted by studies showing that inactivation rather than activation of PVT circuit elements is critical for driving reward-seeking behavior (dynamics that would be missed by gene expression labeling). Specifically, unit recording experiments reveal that a large population of aPVT neurons shows inhibitory responses to sucrose availability (Do-Monte et al., 2017), whereas single-cell calcium imaging experiments show that a large population of pPVT→NAc neurons becomes inhibited during the presentation of sucrose-predictive cues (Reed et al., 2018; Otis et al., 2019). However, in each of these studies, considerable heterogeneity was found as subsets of those PVT neurons also showed excitatory responses to sucrose (Do-Monte et al., 2017) or sucrose-predictive cues (Otis et al., 2019). Despite the heterogeneous activity dynamics among distinct ensembles of PVT neurons, how each of those ensembles might be genetically and functionally distinct is unknown.

Heterogeneous response dynamics in PVT neurons are caused by the integration of signals from a variety of input cell populations. Recently, using two-photon calcium imaging we showed that a majority of prelimbic medial PFC glutamatergic neurons that project to pPVT display inhibitory responses to sucrose-predictive cues (Otis et al., 2017, 2019), although a subset of these inputs also displays excitatory cue responses. Furthermore, using optogenetics we found that inhibitory coding at PFC inputs was critical for downstream cue-evoked inhibition of select pPVT→NAc neurons (Otis et al., 2019). Interestingly, activity in pPVT→NAc neurons was also directed by GABAergic neurons from LHA, which displayed homogeneous excitatory responses during sucrose consumption (Otis et al., 2019). Thus, single pPVT→NAc neurons displayed a multiplexed inhibitory signal during conditioned sucrose seeking that coded for both the cue-reward association (from inhibited PFC glutamatergic inputs) and reward consumption (licking, from excited LHA GABAergic neurons). The encoding dynamics in pPVT are also modulated by other inputs, but the precise nature of such input activity for downstream encoding in projection or genetically-defined neurons is less clear. For example, a smaller population of glutamatergic neurons in LHA that is known to suppress feeding (Stamatakis et al., 2016) also provide input to a subset of pPVT→NAc neurons (Otis et al., 2019), yet how activity in those inputs differs from LHA GABAergic neurons is unknown. Furthermore, GABAergic neurons in the zona incerta (ZI), which form a continuum with the lateral hypothalamus, send a dense projection to pPVT and may become activated during hunger to facilitate feeding through inhibition of pPVT neurons (Zhang and van den Pol, 2017). Activity in a non-canonical dopaminergic input to pPVT from the locus coeruleus also seems to be necessary for bulk excitatory stress responses in pPVT→NAc neurons (Beas et al., 2018), although the precise activity dynamics of these inputs during stress or reward-seeking is unknown. Furthermore, whether the activity at these inputs selectively controls the activity of D2-expressing pPVT→NAc neurons is unknown. Altogether, PVT receives a variety of signals from amino acid and monoamine neurotransmitter inputs that lead to multiplexed activity patterns in PVT that can initiate or prevent the expression of motivated behaviors.

PVT neurons are not just regulated by amino acid and monoamine neurotransmitter inputs, but also by a long list of neuropeptide inputs from the hypothalamus (as reviewed in this thematic series). Despite this knowledge, no studies to date have measured the activity of neuropeptide-specific inputs to PVT *in vivo*. Furthermore, how cell-type-specific neurons in PVT are modulated by these neuropeptides is unclear. Studies that evaluate the activity dynamics of neuropeptide-specific input activity and/or postsynaptic neuropeptide binding in PVT are important for understanding how exactly PVT neurons are engaged to control natural reward and drug-seeking behaviors.

## Novel Approaches to Unraveling PVT Secrets

### Dissociating Cell Types

Due to the multi-faceted heterogeneity among neurons that comprise the PVT (based on projection, gene expression, location, and activity), unraveling the mystery of PVT function for motivated behaviors has remained difficult. Several new approaches could help us untangle, particularly those that emphasize dissection, recording, and perturbation of single neurons. Most importantly, we need a greater resolution for defining unique cell types in PVT, a feat that has been difficult to achieve when evaluating cell populations based on axon projection (due to widespread bifurcation), anatomical location (due to the undefinable gradient that separates each axis of PVT), and activity (due to heterogeneous activity patterns in defined cell populations). Thus, here we propose that scientists prioritize dissection of PVT neurons based on gene expression, as some cell types seem to express completely unique genes and thus are completely isolatable through cre-driver mouse lines and viruses. One approach that would be particularly useful for the field is high-throughput, single-cell mRNA sequencing (e.g., drop-sequencing). Single-cell sequencing could isolate cell-types that express unique genes and/or enriched gene sets (e.g., gene sets that code for opioid receptors or genes that are critical for modulation of hunger), providing unprecedented insight into the cell populations that make up PVT.

### Measuring Activity

There are considerable limitations to experimental approaches that are commonly used for studying PVT activity during or in response to motivated behaviors. One of the most commonly used methods is immediate early gene labeling (e.g., *c-fos*), used in an attempt to show that a particular behavioral event or context activates PVT neurons. Considering that inhibitory rather than excitatory activity dynamics often drive behavior through PVT (see above), conclusions made from such studies should be made with significant caution as such neuronal dynamics could be completely missed by *c-fos* (or Fos) labeling. Another commonly used approach is calcium photometry, used to determine if the bulk cellular dynamics in a single cell population changes over time. This approach is also limiting and possibly misleading, as calcium sensor fluorescence dynamics bias detection of excitatory responses in neurons due to the rapid on but slow off kinetics. As such, in a scenario wherein one cell is inhibited and another cell is excited, bulk photometry is likely to indicate an excitatory response overall. This problem is highly relevant for PVT studies due to heterogeneous activity dynamics found in PVT cell types. Considering this heterogeneity, it is important to perform recordings with single-cell resolution and in identified cell types (e.g., based on gene expression). Calcium imaging or optogenetic photo-tagging with *in vivo* electrophysiology provide the best means to monitor activity in single, genetically-defined neurons (Jennings and Stuber, 2014; Rodriguez-Romaguera et al., 2020) and thus should be considered when studying activity in PVT.

### Resolving Function

Defining the causal function of neuronal activity patterns for behavioral output is a tenant of behavioral neuroscience; however, the neurobiology of PVT makes functional experimentation difficult to apply. Two major issues arising in PVT that are critical to overcome are: (1) inhibitory dynamics in PVT change the nature of “sufficiency” and “necessity” experiments and (2) heterogeneity in PVT makes it difficult to completely dissect the function of discrete neuronal ensembles.

Considering the inhibitory dynamics that often arise during reward consumption and seeking in PVT cell populations (Do-Monte et al., 2017; Otis et al., 2017, 2019), it is important to acknowledge that “necessity” experiments, wherein activity in a particular cell population is inhibited, become problematic. For example, we and others have found that a majority of PVT neurons or PVT→NAc neurons specifically are inhibited during sucrose seeking and consumption (Do-Monte et al., 2017; Otis et al., 2019), and yet inhibition of PVT or specifically PVT→NAc neurons during sucrose seeking did not affect behavior (unless there was a reward omission; Do-Monte et al., 2017). This effect is likely due to the neuronal activity reaching a floor, and thus activation of the cells is truly the experiment that targets “necessity”. Indeed, when Do-Monte et al. (2017) activated PVT→NAc neurons it completely abolished reward-seeking and consumption, suggesting that inhibition of this pathway is necessary for those behaviors. Overall, researchers should be extremely cautious when applying inhibition experiments for ascribing “causality” to PVT and should also consider resolving the ground-truth activity dynamics of studied cell populations before performing such experiments.

The heterogeneous nature of PVT neuronal dynamics has made it difficult to dissect the function of that activity for behavior. For example, recently we found that neurons in the PVT→NAc pathway can be split into two basic categories, neurons that are inhibited and neurons that are excited during the presentation of a sucrose-predictive cue. This heterogeneity, as discussed above, is not unique when studying PVT and should thus be considered for all functional experiments and conclusions. Novel experimental approaches that would be particularly useful involve those that target single neuronal ensembles defined based on activity. This has traditionally been performed through genetic mouse lines that allow “trapping” of activated neuronal ensembles based on immediate-early gene expression (Guenthner et al., 2013; DeNardo and Luo, 2017). However, those methodologies involve highly indirect proxies for activity, lack temporal resolution, and ignore a majority of ensemble dynamics—such as inhibited ensembles—which are critical for modulation of reward-seeking in PVT. Thus, another approach that should be considered is those involving two-photon calcium imaging coupled with single-cell optogenetics for modulation of activity in unique neuronal ensembles defined based on *in vivo* activity patterns. Such experiments would be transformative in that the range of activity dynamics in PVT that contribute to behavior could be resolved.

## Conclusions

Studies described herein reveal the inherent complexity of PVT that seems to be organized as a variety of cell types based on anatomical location, gene expression, connectivity, and *in vivo* activity dynamics—none of which seem to be mutually exclusive. In addition to such heterogeneity, PVT responses during reward-seeking and consumption tend to be inhibitory, making it difficult not only to characterize that activity but also to identify the function of such dynamics for behavior. We propose the use of single-cell transcriptional profiling strategies for defining precise cell types in PVT, as well as excitatory optogenetics to identify how inhibitory activity patterns in these cell types are functionally necessary for controlling behavior. Altogether, studies thus far suggest that PVT influences reward-seeking and consummatory behavior based on a variety of environmental and physiological conditions that amalgamate into multiple PVT output channels to provide integrated decisions over motivated behaviors (Choi et al., 2019). However, due to the inherent complexity of PVT cell types and activity patterns, we are only beginning to understand how PVT serves as a “traffic light” that gates the motivational properties signaling relative safety (GO) vs. risk (STOP) to influence state-dependent reward-seeking behaviors.

## Author Contributions

JM and JO contributed equally to the writing of this review article.

## Conflict of Interest

The authors declare that the research was conducted in the absence of any commercial or financial relationships that could be construed as a potential conflict of interest.
